# The neural coding of tonal working memory load: An functional magnetic resonance imaging study

**DOI:** 10.3389/fnins.2022.979787

**Published:** 2022-10-18

**Authors:** Qiang Li, Dinghong Gong, Huiyi Tang, Jing Tian

**Affiliations:** ^1^College of Education Science, Guizhou Education University, Guiyang, China; ^2^Guizhou Education University, Guiyang, China

**Keywords:** tonal working memory load, SC-MVPA, cortical activation pattern, neural coding, CNN

## Abstract

Tonal working memory load refers to the number of pitches held in working memory. It has been found that different verbal working memory loads have different neural coding (local neural activity pattern). However, whether there exists a comparable phenomenon for tonal working memory load remains unclear. In this study, we used a delayed match-to-sample paradigm to evoke tonal working memory. Neural coding of different tonal working memory loads was studied with a surface space and convolution neural network (CNN)-based multivariate pattern analysis (SC-MVPA) method. We found that first, neural coding of tonal working memory was significantly different from that of the control condition in the bilateral superior temporal gyrus (STG), supplement motor area (SMA), and precentral gyrus (PCG). Second, neural coding of nonadjacent tonal working memory loads was distinguishable in the bilateral STG and PCG. Third, neural coding is gradually enhanced as the memory load increases. Finally, neural coding of tonal working memory was encoded in the bilateral STG in the encoding phase and shored in the bilateral PCG and SMA in the maintenance phase.

## Highlights

– The neural coding differences between tonal working memory and the control condition was detected in the bilateral STG, PCG, and SMA.
– Neural coding differences between different tonal working memory loads were detected in the bilateral STG and PCG.
– Neural coding is enhanced as the memory load increases.
– Neural coding of tonal working memory was encoded in the bilateral STG and stored in the bilateral PCG and SMA in the maintenance phase.

## Introduction

Working memory describes the ability to temporally maintain and manipulate information in the mind and is important for high-level cognitive functions such as reasoning and decision-making (Baddeley, 2003, 1992). In the auditory working memory domain, verbal and tonal working memory have attracted much attention because of their association with the cognition of language and music (Schulze and Koelsch, 2012; Kaiser and Brosch, 2016). During the past two decades, the neural basis of verbal and tonal working memory has been well studied with numerous neuroimaging methods, such as functional magnetic resonance imaging (fMRI) (Linke et al., 2011; Schulze et al., 2011), electro-/magnetoencephalography (EEG/MEG) (Leiberg et al., 2006; Guimond et al., 2011), local field potential (LFP) (Bigelow et al., 2014; Yu et al., 2021), and functional near-infrared spectroscopy (fNIRS) (Jeong and Ryu, 2016; Rovetti et al., 2021).

In verbal working memory studies, it has been found that Broca’s area, premotor areas (PMC), the STG, the inferior parietal lobule (IPL), the superior parietal lobule (SPL), insula, and the cerebellum are involved in the processing of verbal working memory (Paulesu et al., 1993; Awh et al., 1996; Fiez et al., 1996; Bamiou et al., 2003; Gruber and von Cramon, 2003; Crottaz-Herbette et al., 2004; Ravizza et al., 2004; Chen and Desmond, 2005; Kirschen et al., 2005; Koelsch et al., 2009; Cowan et al., 2011; Huang et al., 2013; Li et al., 2014; Fegen et al., 2015; Majerus et al., 2016; Emch et al., 2019; Ghaleh et al., 2020; Hoddinott et al., 2021). In the tonal working memory domain, relevant activations were found in the intra parietal sulcus (IPS), inferior frontal gyrus (IFG), cerebellum, dorsal lateral prefrontal cortex (DLPFC), supramarginal gyrus (SMG), STG, insula, and hippocampus (Zatorre et al., 1994; Holcomb et al., 1998; Griffiths et al., 1999; Gaab et al., 2003; Foster and Zatorre, 2010; Linke et al., 2011; Schulze et al., 2011; Albouy et al., 2013, 2015, 2017; Foster et al., 2013; Kumar et al., 2016; Czoschke et al., 2021; Erhart et al., 2021). Despite the differences, these studies converged to form a consensus that verbal and tonal working memory share a similar frontoparietal network as neural basis (Schulze and Koelsch, 2012).

Working memory load refers to the number of items held in working memory (Nolden et al., 2013). The neural basis underlying working memory load has been widely studied in the visual and verbal domains. By testing BOLD signal strength (Cowan et al., 2011) found that the left IPS had load-dependent activity for both verbal and visual working memory. In a visual change detection study with monkeys, (Pinotsis et al., 2019) modulated the number of objects (1–3 objects) remembered by monkeys and found that changes in working memory load influenced the connections between the lateral intraparietal area (LIP), prefrontal cortex (PFC), and frontal eye field (FEF). In an fMRI study distinguishing the neural basis between verbal working memory load and attention, (Huang et al., 2013) found that activities in the DLPFC, SMA, IPL, SMG, and right anterior insula increased with increasing verbal working memory load. When distinguishing the neural basis of verbal working memory load and rehearsal rate, (Fegen et al., 2015) manipulated the memory load and rehearsal rate and designed a long 45 s delay period. They found that during early delay (2–16 s) and middle delay (16–30 s), activities in the IFG, PMC, middle frontal gyrus (MFG), and SPL were linearly correlated with memory load. Using fMRI and a delayed visual letter recognition task, Zarahn et al. (2005) found that BOLD signals in the precentral gyrus (PCG), MFG, IPL, SPL, and some other areas showed a linear relationship with memory load. In addition to these studies, some other studies (Nystrom et al., 2000; Ravizza et al., 2004; Todd and Marois, 2004; Todd et al., 2005; Xu and Chun, 2006; Majerus et al., 2012) also reported load-related neural activity when studying verbal and visual working memory.

In recent years, the neural encoding of different memory loads has begun to attract researchers’ attention in the verbal and visual domains. In a decoding study (Majerus et al., 2016) of verbal and visual working memory, different memory loads were found to have distinguishable neural coding in the superior frontal and posterior parietal regions of the dorsal attention network. In an fMRI study (Weber et al., 2016) studying the interaction between working memory load and precision of working memory, memory load-related neural coding was found in the superior IPS. In a decoding study (Bååth, 2009) using an artificial neural network, the PFC was found to carry verbal working memory load-dependent neural coding.

Relatively, the amount of research on tonal working memory load is small and even less for decoding research. Using fNIRS (Tseng et al., 2018) studied neural correlates of tonal working memory load in high-anxiety participants and patients. A stronger signal was observed in the right orbital prefrontal and ventrolateral cortex when attending to high load tonal stimuli (Guimond et al., 2011; Lefebvre et al., 2013). Studied tonal working memory with EEG and found that the amplitude of a component named the sustained anterior negativity increased as the tonal working memory load increased. In an MEG study, using pure tones as acoustic materials (to minimize non-tonal working memory activity), (Grimault et al., 2014) found that response amplitude in parietal, frontal, and temporal cortices increased with increasing tonal working memory load. In another MEG study, with one or two tones as stimuli (Nolden et al., 2013) found that right parietal structures, IFG, inferior temporal gyrus and bilateral STG showed stronger activity when maintaining higher tonal working memory load. To our knowledge, no decoding study has been done to tonal working memory load.

In this study we focused on the neural coding differences between different tonal working memory loads. We proposed a surface space and CNN-based MVPA method to better study this problem. Carrying out MVPA in surface space has some advantages. First, analyzing in surface space can avoid noise from white matter and other non-gray matter tissues. Second, for areas located near the longitudinal fissure, analyzing in surface space can avoid interference from the other hemisphere. Third, neural coding is easier to display in surface space. In addition to the choice of space, which classifier to choose is also important. When implementing MVPA in surface space, the searchlight (local pattern space) is a curved surface, which can be easily flattened into a 2D image. As one of the most shining technologies in image recognition (Gu et al., 2018), CNN is an ideal classifier for 2D image classification, and is thus suitable for the surface space based MVPA method. Using this SC-MVPA method, we found that the neural coding of different tonal working memory loads was different in the bilateral STG and PCG.

## Materials and methods

### Subjects

We recruited 23 student participants (12 male, age range 18–23, right handed) at Southwest University. Participants reported that they had normal hearing and did not have absolute pitch. Except for general school education, subjects had no extra music training. Subjects signed informed consent forms and were paid approximately 20 dollars for their participation. The local ethics committee of Southwest University approved this experimental protocol (item number: H21053).

### Experimental paradigm

**FIGURE 1:**
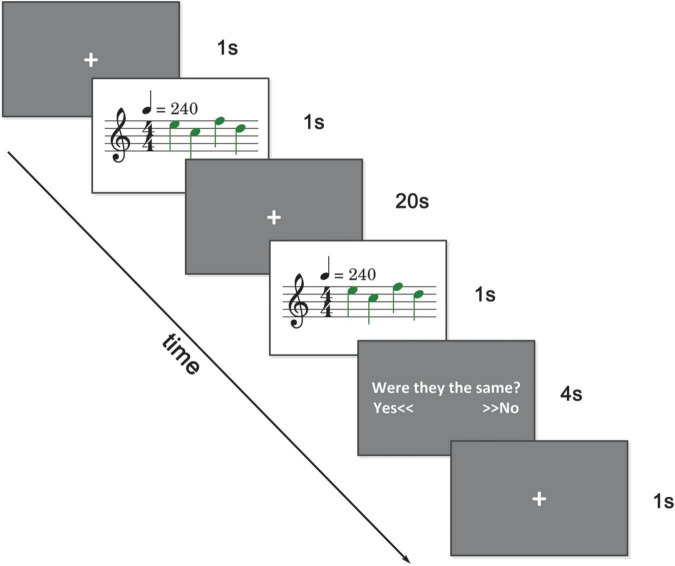
Experimental paradigm. A series of tones (1–4 tones) were presented and after 20 s, another series of tones (same in length) were presented. There was a 50 percent chance that there was a tone change. Subjects were asked to answer whether these two series of tones were the same within 4 s.

A delayed match-to-sample paradigm was implemented accompanied by fMRI scanning. As illustrated in Figure 1, a trial began with 1 s of quietness. After that, a series of piano tones (1-4 tones, randomly selected), i.e., the sample, were presented to the subjects. After a 20 s delay, another series of tones (same length) were presented as the probe. In 50% chance, one tone (in random position) of the probe was changed in two natural tones. Subjects had 4 s to answer whether the sample and the probe were the same with two buttons. Twelve subjects pressed the buttons with the left index finger, while 11 subjects pressed the buttons with the right index finger. Materials of tones were selected according to the key of C major from C1 to D2. To minimize interference from other musical aspects, such as rhythm and tempo, all stimuli materials were 4/4 meters and 240 bpm. Tones were presented through headphones. We set up a BOLD scanning run (1 min, after T1 scanning, to simulate the experimental environment) to adjust the system volume. A piece of music was played in this run and subjects were asked to adjust the system volume as large as possible on the premise of feeling comfortable. The sound level of this music and the tones were normalized with Adobe Audition CS6.^1^

During the first second and the 20 s delay period, subjects were asked to focus on the central cross on the screen. There were 100 trials in total, with 20 trials for each experimental condition (load 1–4) and 20 trials for the control condition (load 0). In the control condition, no tone was presented and subjects were asked to press the button represent the same in the answering period. The control condition mainly served as a comparison and baseline in the data analysis. Trials were presented in random sequence. There were five scanning runs in total. Delayed match-to-sample tasks were implemented in the last three runs, with 33 trials in the third and fourth runs and 34 trials in the fifth run. Subjects were trained with a simulation experiment before entering the scanning room.

### Functional magnetic resonance imaging data acquisition

We acquired structural (T1-weighted) and functional BOLD images with a 3T Siemens Prisma_fit scanner. Structural data were acquired first, followed by 4 runs of BOLD data acquisition. The parameters of structural scanning were as follows: Resolution (1 × 1 × 1 mm^3^), TR (2,530 ms), TE (2.98 ms), TI (1,100 ms), flip angle (7 deg), acceleration factor PE (2), slice per slab (192), FoV read (256 mm). The parameters of functional scanning were as follows: Resolution (2.5 × 2.5 × 2.5 mm^3^), TR (1,000 ms), TE (30 ms), flip angle (73 deg), acceleration factor slice (4), slices (56), FoV read (195 mm).

### Data preprocessing

fMRI data were preprocessed with FreeSurfer.^2^ Preprocessing included two stages, and each stage corresponded to an encapsulated function offered by FreeSurfer. The first stage was cortical reconstruction using structural data. This stage includes many steps, including motion correction and conform, non-uniform intensity normalization, Talairach transform computation, intensity normalization, skull strip, remove neck, white matter segmentation, tessellation, smooth, inflate, spherical mapping, spherical registration, cortical parcellation (labeling), cortical parcellation mapped to ASeg and some other steps. Although very complicated, all these steps were encapsulated in the function “recon-all,” and researchers can easily implement this stage by calling this function. The second stage was preprocessing the functional data. This stage included steps such as registration template creation, motion correction, slice-timing correction, functional-anatomical registration, mask creation, intensity normalization, resampling raw time series to mni305, lh, and rh surface space, spatial smoothing (only for general liner model (GLM) analysis, MVPA uses data without spatial smoothing) and some other steps. All these steps were encapsulated in the function “preproc-sess.” By preprocessing, functional data were resampled into surface space (lh, rh) and a mni305 2 mm template. Common MVPA analysis is generally conducted at a resolution of approximately 3 mm (Lee et al., 2011; Linke and Cusack, 2015; Uluç et al., 2018). To be consistent with these studies and reduce the amount of calculation, we downsampled the preprocessed volume data into a mni305 3 mm template using the function “reslice_nii,” which was offered by NIFTI.^3^

### SC-MVPA

**FIGURE 2:**
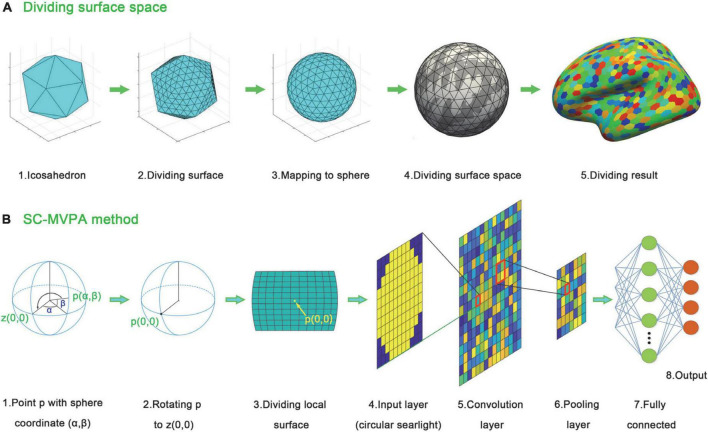
Analyzing stream of SC-MVPA. **(A)** Dividing algorithm used to divide the template surface space. **(B)** Analyzing stream of SC-MVPA. For any point p (α,β) in the downsampled sphere space of fsaverage, the sphere is rotated to rotate p to z (0,0). After that an 11 × 11 grid around p (0,0) is acquired. The BOLD signal in cells are averaged, forming an 11 × 11 searchlight. A circle (with a radius of 11/2) is applied to the 11 × 11 grid, forming a circular searchlight. The circular searchlight then entered into the CNN.

To better analyze and display the local neural coding of tonal working memory loads, we proposed an SC-MVPA method in this paper. The analysis pipeline of SC-MVPA is illustrated in Figure 2. SC-MVPA implemented analysis in the spherical space offered by FreeSurfer. This space has 163842 vertices for each hemisphere. The unfolded human cerebral cortex (each hemisphere) has a total surface area of approximately 0.12 square meter (Toro et al., 2008). Thus, each vertex occupies an area of approximately 0.73 mm^2^. Considering the thickness of the cerebral cortex of 2-4 mm (Ribeiro et al., 2013), each vertex occupies a volume of approximately 2 mm^3^, which is approximately an order of magnitude smaller than the resolution of the original functional data (2.5 × 2.5 × 2.5≈15.6 mm^3^). If SC-MVPA was directly implemented in the original spherical space, it would cause repetitive calculation and increasing amount of calculation. Thus, the first step of SC-MVPA is downsampling the spherical template space. As illustrated in Figure 2A, we first constructed an icosahedron. The edges of the icosahedron were equally divided into 40 sections. The points on adjacent edges were connected and formed new points of intersection. The center of the icosahedron was connected with these points, and the connections were projected onto the surface of the circumscribed sphere of the icosahedron. Based on this method, a total of 16,002 points were on the circumscribed sphere. These points were projected to the template spherical space and formed a downsampled spherical space.

For each point on the downsampled spherical space, the following operations were performed. As illustrated in Figure 2B, assuming that the spherical coordinate of a point *p* was (α,β), the sphere was rotated to move point *p* to point *z* (0,0). Then, around point *p* (0,0), an 11 × 11 grid was constructed along the longitude and latitude (1/35 radian per cell). The BOLD signal of vertices in each cell was averaged and formed an 11 × 11 image. A circular searchlight template (with a radius of 5.5) and z-score normalization were applied to this image. The normalized image is then entered into a CNN as input data. This CNN consisted of an input layer (11 × 11), a convolution layer (11 × 11, padding 11), a relu layer, a max pooling layer (2,2), a fully connected layer (equal to the number of categories to be classified), a softmax layer and a classification layer. There were three experimental runs in total. A threefolded cross validation, with two runs serving as the training data and one run serving as the classifying data, was performed. The recognition rates of these three times were averaged and entered into the group level analysis.

In group-level analysis, the recognition rate across subjects of each point was compared with chance probability with a one-sample *t*-test (right tail). Multicomparison correction was performed using non-parameter permutation tests (Nichols and Holmes, 2002; Oosterhof et al., 2010; Czoschke et al., 2021; Erhart et al., 2021). The permutation simulation tests simulate the process of generating active clusters from random noise. To simulate clusters generated from random noise, the recognition rate of each subject and point was subtracted by the chance probability. The sign of the resulting differences was randomly flipped. These randomly flipped results constitute random noise. Then, the flipped differences across subjects of each point were compared with zero with a one-sample *t*-test (right tail). This process was permutated 10,000 times. For each permutation, the size of the largest cluster was recorded. If the size of the active cluster is larger than 95% of the simulated clusters, the probability that the active cluster is generated by random noise is less than 5% [corresponding to a familywise error rate (FWE) of 0.05]. The sizes of the largest simulated clusters were sorted (from large to small), and the size of the 500th largest cluster was recorded as a criterion. The cluster sizes of the group-level analysis results were compared with the size of the criterion, and only the clusters larger than the criterion were reported (corresponding to FWE < 0.05). All these SC-MVPA steps were implemented in MATLAB 2022a.^4^

### Training and classification

**FIGURE 3:**
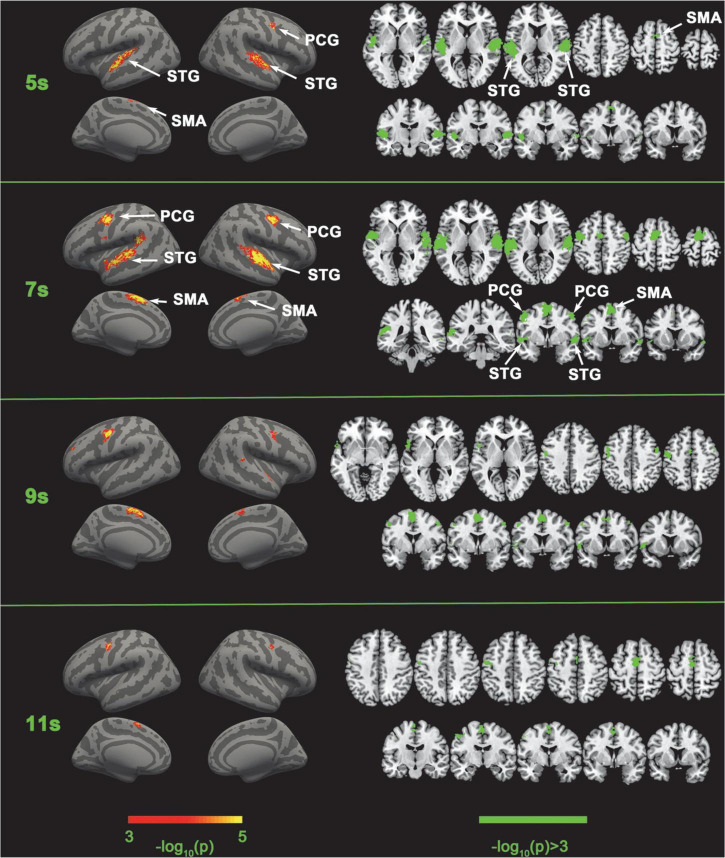
Comparison of SC-MVPA and the traditional volume space-based MVPA method. The significance of the classification of the five categories of SC-MVPA is shown in the left part, and traditional MVPA is shown in the right part.

**FIGURE 4:**
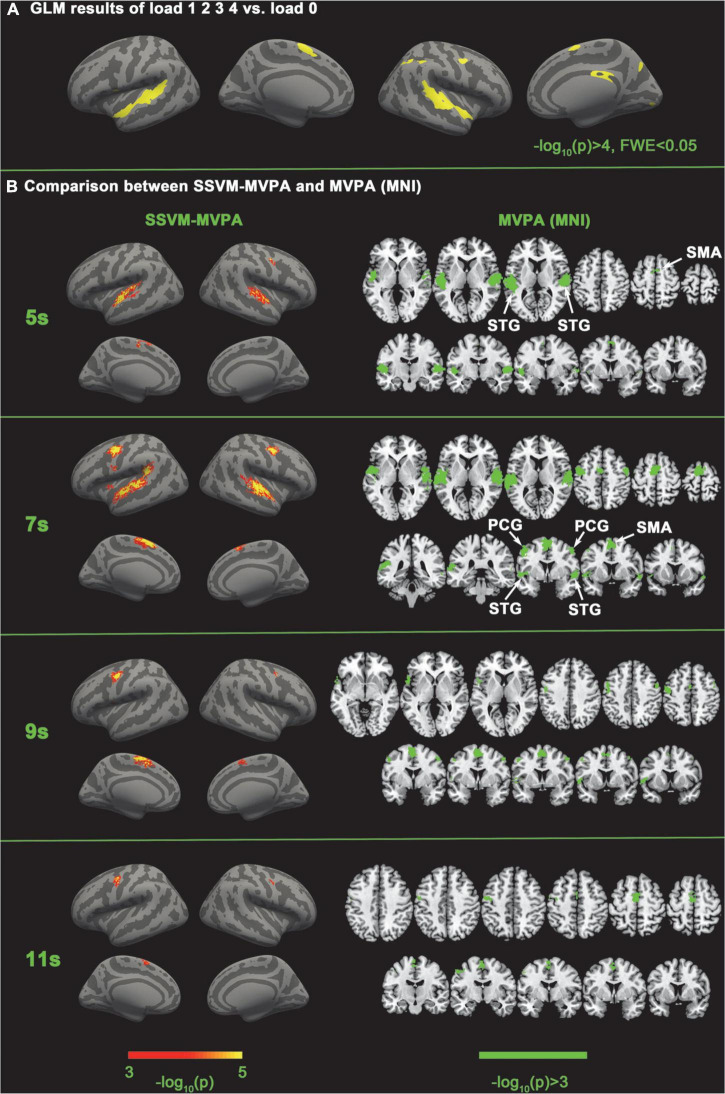
GLM results of load 1 2 3 4 vs. load 0 and comparison between SSVM-MVPA and volume space-based MVPA. **(A)** Traditional contrast analysis between the experimental conditions and the baseline condition. **(B)** Comparison between SSVM-MVPA and MVPA (MNI). The figures of MVPA (MNI) are the same as Figure 3.

**FIGURE 5:**
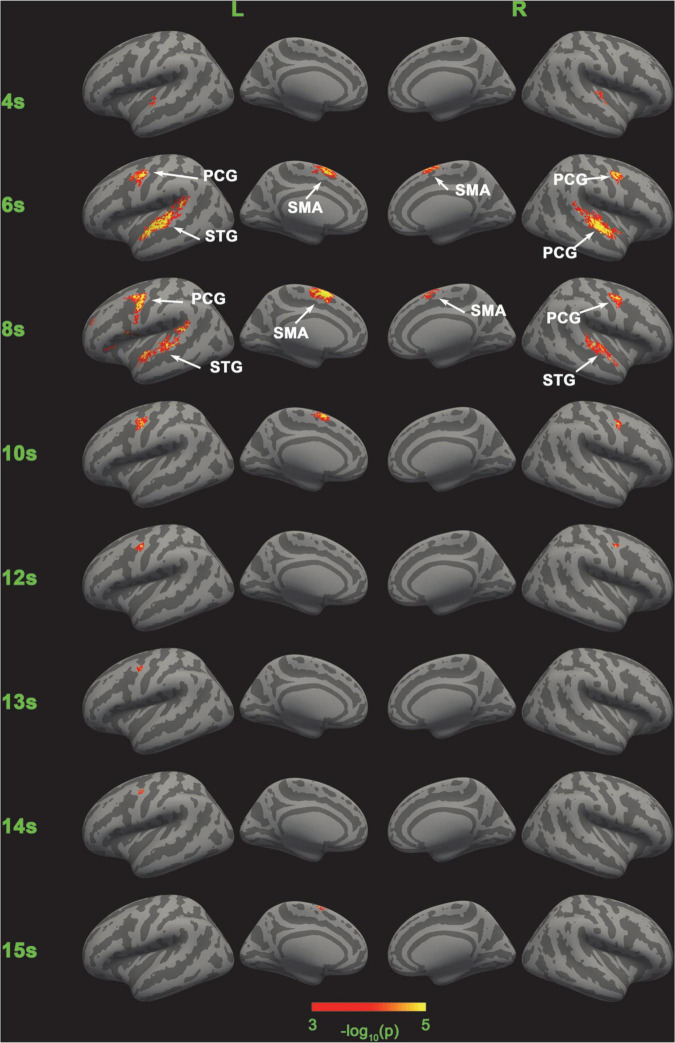
Significance of the classification of the five categories of SC-MVPA. The neural coding of the five conditions are distinguishable in bilateral STG in 4, 6, and 8 s, in left SMA in 6, 8, 10, and 15 s, in right SMA in 6 and 8 s, in left PCG in 6, 8, 10, and 12∼14 s, and in right PCG in 6, 8, 10, and 12 s.

We first implemented five-category classification analysis, during which all conditions (load 0–4) entered the training and classification process. The main purpose of the five-category classification was to first, test whether there was a neural coding difference underlying these five conditions; second, make a comparison between SC-MVPA and the traditional MVPA method; and third, observe the results of classification and analyze the extent to which these five conditions differ on neural coding. A threefolded cross validation, with two runs serving as the training data and one run serving as the classifying data, was performed. There are 66∼67 trials in the training set and 33∼34 trials in the testing set. The training parameters of the CNN of the five-category classification were solver name (“sgdm”), max epochs (400), and initial learning rate (0.01). In the group-level analysis, the recognition rate was compared with 20% (significance *p* < 0.001, FWE < 0.05). In the five-category classification, we also implemented a traditional volume space-based MVPA (MNI) to make a comparison. This method is based on volume space and a support vector machine (SVM), using a spherical searchlight with a radius of three voxels (Lee et al., 2011). The threefolded cross validation and FWE correction were the same as those for SC-MVPA. Besides, we also performed the surface space- and SVM-based MVPA (SSVM-MVPA) for five-category classification. The classifier of SC-MVPA is replaced with SVM, and other settings remain unchanged. For comparison, the results of SC-MVPA and traditional MVPA (5, 7, 9, 11 s) are shown in Figure 3, the results of SSVM-MVPA and traditional MVPA (5, 7, 9, 11 s) are shown in Figure 4B. The remaining results of SC-MVPA are shown in Figure 5. The coordinates of Figure 3 are shown in Tables 1, 2; the coordinates of Figure 4 are shown in Table 3.

**TABLE 1:** Results of volume space based MVPA (Figure 3 right).

Time	Peak intensity	Number of voxels	Region	MNI coordinates		
				
				x	y	z
5s	9.6	575	Superior temporal gyrus left	–55	–16	4
	8.0	431	Superior temporal gyrus right	53	–19	10
	6.2	70	Supplement motor area	–7	–1	61
7s	8.3	642	Superior temporal gyrus right	59	–12	4
	7.5	870	Superior temporal gyrus left	–52	–7	7
	7.4	314	Precentral gyrus left	–52	2	43
	6.2	134	Precentral gyrus right	50	–1	46
	4.6	56	Superior parietal lobule left	–25	–70	49
	7.6	444	Supplement motor area	–4	–7	64
9s	6.0	118	Superior temporal gyrus left	–58	20	–5
	5.8	215	Precentral gyrus left	–37	5	40
	6.2	75	Precentral gyrus right	47	–1	49
	8.4	354	Supplement motor area	–2	–2	62
11s	5.6	120	Precentral gyrus left	–43	–13	49
	5.6	166	Supplement motor area	–1	–7	52

**TABLE 2:** Results of SC-MVPA (Figure 3 left).

Time	Peak intensity	Size of cluster (mm^2)	Region	Talairach coordinates		
				
				x	y	z
5s	7.9	1,552	Superior temporal gyrus left	–43	–23	–2
	6.4	1,444	Superior temporal gyrus right	62	–6	–2
	4.2	170	Supplement motor area left	–6	20	55
	4.4	85	Precentral gyrus right	50	–1	44
7s	9.2	1,408	Superior temporal gyrus right	50	–13	3
	7.3	1,888	Superior temporal gyrus left	–54	–10	1
	8.6	734	Precentral gyrus left	–44	4	41
	4.0	48	Precentral gyrus left	–58	5	20
	7.5	465	Precentral gyrus right	46	–1	44
	9.0	876	Supplement motor area left	–11	9	60
	6.2	340	Supplement motor area right	11	9	61
9s	6.2	101	Superior temporal gyrus right	52	–9	–2
	7.4	468	Precentral gyrus left	–53	–4	39
	5.2	371	Precentral gyrus right	50	2	41
	6.6	624	Supplement motor area left	–8	13	51
	4.1	74	Rostral middle frontal gyrus left	–35	38	24
	5.3	289	Supplement motor area right	8	10	53
	4.5	90	Superior temporal gyrus right	63	–35	10
11s	6.2	436	Precentral gyrus left	–48	–2	43
	6.3	241	Supplement motor area left	–6	7	59
	4.6	153	Precentral gyrus right	46	1	42

**TABLE 3:** Results of SSVM-MVPA and GLM analysis.

Time/GLM	Peak intensity	Size of cluster (mm^2)	Region	Talairach coordinates		
				
				x	y	z
5s	8.5	1,227	Superior temporal gyrus left	–43	–23	–2
	6.4	985	Superior temporal gyrus right	52	–9	–2
	5.4	228	Supplement motor area left	–6	15	56
	6.2	110	Precentral gyrus right	50	–3	44
7s	7.0	1,648	Superior temporal gyrus right	60	–16	–1
	9.2	2,379	Superior temporal gyrus left	–54	–10	1
	8.9	685	Precentral gyrus left	–51	–2	43
	4.7	90	Precentral gyrus left	–58	–1	15
	4.2	81	Insula left	–37	–2	4
	7.1	451	Precentral gyrus right	46	2	41
	7.8	875	Supplement motor area left	–6	8	60
	5.5	307	Supplement motor area right	12	9	59
9s	7.4	497	Precentral gyrus left	–52	–4	42
	4.9	58	Precentral gyrus right	43	4	40
	7.3	725	Supplement motor area left	–8	–6	55
	4.5	167	Supplement motor area right	8	6	56
11s	5.6	282	Precentral gyrus left	–50	–3	44
	5.2	151	Supplement motor area left	–6	6	60
	4.5	60	Precentral gyrus right	52	2	38
GLM results	9.6	1,757	Superior temporal gyrus left	–49	–3	–13
	11.2	379	Supplement motor area left	–7	0	62
	7.1	112	Inferior frontal gyrus left	–51	6	0
	11.7	2,098	Superior temporal gyrus right	48	–6	–14
	8.2	161	Supplement motor area right	7	4	64
	6.4	164	Supramarginal gyrus right	49	–42	42
	6.0	332	Inferior parietal gyrus right	39	–65	47
	5.8	139	Precuneus right	12	–67	35
	5.8	112	Lingual gyrus right	5	–90	–9
	5.7	169	Precentral gyrus right	51	–1	45
	5.3	176	Posterior cingulate gyrus right	4	–19	28

We then implemented four-category classification (load 1-4, SC-MVPA) to explore whether the neural coding of different tonal working memory loads was distinguishable. The training parameters were the same as in the five-category classification. The three-folded cross validation and FWE correction were the same as those for five-category classification. Since the stimuli were presented randomly, the size of the training and test sets varied. The results of the four-category classification showed that no brain area’s recognition rate was significantly higher than the chance level (25%, significance *p* < 0.001, FWE < 0.05).

**FIGURE 6:**
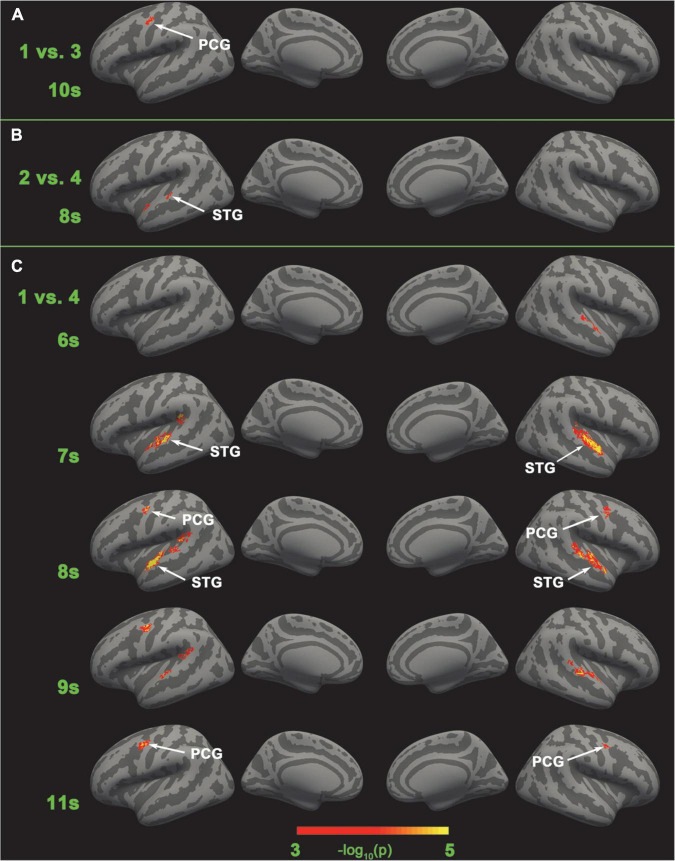
Brain areas that have significant different neural coding for different tonal working memory loads. No brain area has significant different neural coding for adjacent tonal working memory loads. **(A)** The left PCG has significant different neural coding for load 1 vs. load 3 in the 10th second. **(B)** The left STG has significant different neural coding for load 2 vs. load 4 in the 8th second. **(C)** The left STG has significant different neural coding for load 1 vs. load 4 in the 7∼9 s. The right STG has significant different neural coding for load 1 vs. load 4 in the 6∼9 s. The bilateral PCG has significant different neural coding for load 1 vs. load 4 in the 8∼11 s.

We also performed two-category classification for adjacent memory loads (i.e., load 1 vs. load 2, load 2 vs. load 3, load 3 vs. load 4) to see whether neural coding of adjacent memory loads was distinguishable. As the categories to be classified were decreased to two kinds, the max epochs were adjusted to 100, and the group-level *t*-test was compared with 50% (significance *p* < 0.001, FWE < 0.05). After the adjacent load classification, we also performed two-category classification for nonadjacent memory loads (i.e., load 1 vs. load 3, load 2 vs. load 4, and load 1 vs. load 4) to determine whether neural coding of non-adjacent loads was distinguishable. The threefolded cross validation and FWE correction were the same as those for five-category classification. Since the stimuli were presented randomly, the size of the training and test sets varied. Parameters in non-adjacent load analysis were the same as in adjacent load analysis. The classification results of non-adjacent loads are shown in Figure 6.

### Local neural coding of tonal working memory loads

**FIGURE 7:**
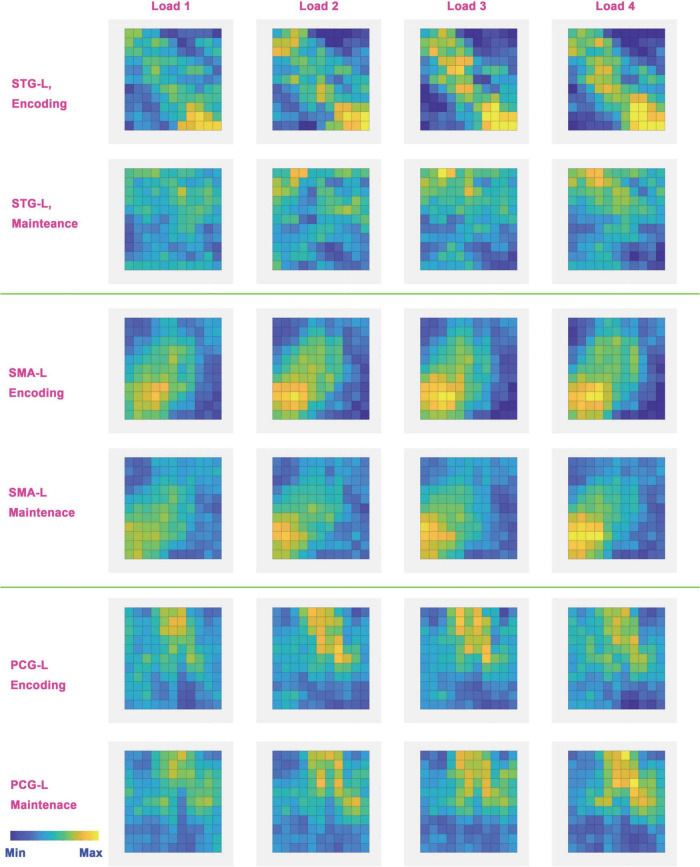
Neural coding (local BOLD signal pattern) in the left STG, PCG, and SMA. These neural codes were obtained by subtracting the neural codes of the baseline condition from the neural codes of each load. Considering the 4∼6 s delay of BOLD signal, the neural codes of the encoding phase were acquired from the 6th second and the neural codes of the maintenance phase were acquired from the 12th second. Bright colors indicate a strong BOLD signal and dark colors indicate a weak BOLD signal.

To further analyze the neural coding of tonal working memory loads, we averaged the BOLD signal of three searchlights (STG-L, SMA-L, PCG-L) of each condition across trials and subjects. The three searchlights (without applying a circular searchlight) were selected according to the peak locations of the five-category classification results in 7 s. The averaged local neural coding of loads 1-4 was subtracted by the averaged local neural coding of the control condition and is displayed in Figure 7. Considering the 4–6 s delayed effect (Baumann et al., 2010) of the BOLD signal, we chose the local neural coding of 6 and 12 s as the representations of the encoding and maintenance phases.

### General liner model analysis

To reveal the common activated regions of tonal working memory, we performed the traditional GLM analysis for load 1 2 3 4 vs. load 0 with FreeSurfer. The stimulus onset times were convolved with a standard hemodynamic response function curve and entered the GLM analysis. The head motion parameters also entered the GLM analysis. The vertex-wise threshold was set to –log_10_(p) > 3 and the family-wise threshold was set to FWE < 0.05. The GLM results are shown in Figure 4A and Table 3.

## Results

### General liner model results

As shown in Figure 4A and Table 3, tonal working memory activate the bilateral STG, SMA, left Inferior frontal gyrus, and right PCG, lingual gyrus, precuneus gyrus, and SMG.

### SC-MVPA vs. traditional MVPA

Five-category classification results of SC-MVPA and the traditional MVPA of 5, 7, 9, and 11 s are shown in Figure 3. The time (e.g., 5 s, 9 s) represented which frame of the BOLD data was analyzed (the first second corresponded to the second the sample was presented). SC-MVPA and traditional.

MVPA showed highly consistent classification results. For example, both methods showed that in 5 and 7 s, neural coding in the bilateral STG was distinguishable and in 7 and 9 s, neural coding in bilateral PCG were distinguishable. Meanwhile, there were some differences in detail for these two methods. At 5 and 11 s, SC-MVPA showed that only the left SMA showed distinguishable neural coding; but in traditional MVPA, it can be seen that both the bilateral SMA and the longitudinal fissure were marked as distinguishable. It could also be found that using SC-MVPA, in 5 and 11 s, the right PCG showed distinguishable neural coding, but traditional MVPA failed to detect the neural coding difference in this area.

### Five-category classification results of SC-MVPA

**FIGURE 8:**
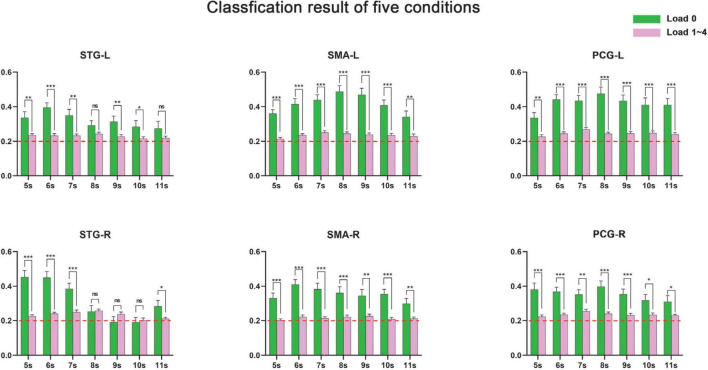
Accurate rates (number of correctly identified trials/total number of trials) of the classification of the five categories. The accurate rates of load 0 in 5∼11 s are significantly higher than other conditions. *Means *p* < 0.05; ^**^means *p* < 0.01; and ^***^means *p* < 0.001.

Figures 3, 5, 8 show that there were distinguishable neural coding differences for the five conditions in the bilateral STG, PCG, and SMA. To be specific, distinguishable neural coding differences can be found in the bilateral STG in 4–8 s, in the right STG in 9 s, in the bilateral PCG in 6–11 s, in the right PCG in 5 s, in the left PCG in 13–14 s, in the bilateral SMA in 6–9 s, and in the left SMA in 5, 10, 11, and 15 s.

The detailed recognition rates (number of correctly identified trials/total number of trials) of the control condition and loads 1–4 are displayed in Figure 8. It can be seen that the recognition rate of the control condition was significantly higher than the averaged recognition rate of loads 1–4, while the averaged recognition rate of loads 1–4 was just slightly higher than the chance level (20%). In addition, some areas (e.g., SMA-L, PCG-L) showed significant distinguishable neural coding after 8 s, while neural coding in right STG was hard to distinguish after 8 s.

### Five-category classification results of SSVM-MVPA

SSVM-MVPA obtained consistent results with SC-MVPA when performing five classifications. The difference is that at the 7th second, SSVM-MVPA detected neural coding differences in the left insula but SC-MVPA did not, and at the 9th second, SC-MVPA detected neural coding differences in the right STG but SSVM-MVPA did not.

### Four-and two-category classification results

SC-MVPA showed that no brain area’s neural coding was distinguishable in the four-category classification and adjacent load classification. However, in non-adjacent load classification analysis, distinguishable neural coding was found in the bilateral STG and PCG. Specifically, distinguishable neural coding was found in 10 s in the left PCG (load 1 vs. load 3), 8 s in the left STG (load 2 vs. load 4), and 6–11 s in the bilateral STG and PCG (load 1 vs. load 4). The results of non-adjacent load classification can be found in Figure 6.

### Local neural coding of tonal working memory

Overall, different memory loads shared similar neural coding in the left STG, PCG, and SMA in the encoding and maintenance phases. One exception is STG-L, the neural coding in which disappeared in the maintenance phase. In addition, neural coding showed a tendency to enhance with increasing memory load. In the encoding phase, the local neural coding of STG-L was obviously enhanced as the memory load increased. A similar tendency also existed in SMA-L and PCG-L in both the encoding and maintenance phases. The exception was also in STG-L because the local neural coding disappeared in STG-L in the maintenance phase.

## Discussion

### SC-MVPA method

In this paper, the SC-MVPA method has been shown to be a feasible fMRI data analysis method. By comparison, we found that SC-MVPA has higher spatial resolution and a higher capability of detecting subtle neural coding differences compared with traditional volume space and SVM-based MVPA. SC-MVPA showed that only the left SMA exhibited distinguishable neural coding in the five-category classification in 5 and 11 s, while traditional MVPA showed that both the bilateral SMA and the longitudinal fissure had distinguishable neural coding. We argue that this is because for areas located near the longitudinal fissure, traditional MVPA is easily influenced by signals from the other hemisphere. Therefore, SC-MVPA has better spatial resolution than traditional MVPA in areas located near the longitudinal fissure. In addition, SC-MVPA successfully detected the neural coding difference in the right PCG in the five-category classification in 5 s and 11 s, while traditional MVPA failed to detect this area. This result indicated that SC-MVPA has a higher ability to detect subtle neural coding differences than traditional MVPA. Besides, the results of SSVM-MVPA are consistent with those of SC-MVPA, which indicates that the advantage of SC-MVPA is not obtained by classifier, but the acquisition method of searchlight improves the spatial positioning accuracy of SC-MVPA.

### Five-category classification

The classification results of the five categories showed that there were distinguishable neural coding differences in the bilateral STG, PCG, and SMA for the five conditions. However, this does not mean that the neural coding of different memory loads (loads 1–4) was distinguishable in these areas. By analyzing the recognition rate of each condition, we found that the recognition rate of the control condition was significantly higher than the averaged recognition rate of loads 1–4, while the averaged recognition rate of loads 1–4 was only slightly higher than the chance level. We argue that these results indicated that first, the neural coding of the control condition was significantly different from that of loads 1–4 so that it was easy to distinguish the control condition from loads 1–4 and hence acquired a high recognition rate of the control condition; second, the neural coding of loads 1–4 was similar to each other so that it was difficult to distinguish the neural coding of different memory loads and that the average recognition rate of loads 1–4 was low.

This view was supported by the classification results of four categories and adjacent loads. The four- category classification showed that the neural coding of loads 1–4 were not distinguishable. Classification of adjacent loads showed that the neural coding of adjacent loads was also not distinguishable. In addition, the neural coding shown in Figure 7 also showed that different tonal working memory loads have similar neural coding. All these results supported the view that neural coding of different memory loads was similar and difficult to distinguish.

Thus, it can be concluded that this was because the classifier successfully detected the difference between the control condition and memory loads (rather than distinguishing memory loads from each other) so that the recognition rate of the bilateral STG, PCG, and SMA was significantly higher than the chance probability. This conclusion was supported by Linke et al. (2011), Kumar et al. (2016), and Uluç et al. (2018), who also found that the bilateral STG, PCG, and SMA showed distinguishable neural coding between the control condition and tonal working memory. Thus, the classification results of the five categories mainly represented the neural coding difference between the control condition and tonal working memory rather than the difference between memory loads.

### Classification of non-adjacent loads

The classification results of loads 1–4 and adjacent loads showed that the neural coding of tonal working memory loads was not distinguishable. However, classification of non-adjacent loads showed that neural coding was distinguishable in the left PCG (10 s, load 1 vs. load 3), left STG (8 s, load 2 vs. 4), and bilateral STG and PCG (6–11 s, load 1 vs. 4). These results may seem contradictory, but they are actually explicable. One reasonable explanation is that neural coding of adjacent loads was indeed hard to distinguish, but as the load difference increases, the difference between neural coding also increases and becomes easy to distinguish. This explanation can explain why neural coding of adjacent loads was not distinguishable and why neural coding difference between load 1 vs. load 4 was more obvious than that of load 1 vs. load 3 and load 2 vs. load 4 (as shown in Figure 6). Beyond that, the neural coding shown in Figure 7 indeed showed a gradual enhancing process and thus supported this explanation. Furthermore, this explanation can explain why neural coding in four categories classification were not distinguishable—because neural coding of adjacent loads were too similar and would interfere with the recognition of each other.

### Neural coding of tonal working memory loads

Partial neural coding of tonal working memory in the encoding and maintenance phases is shown in Figure 7. Some meaningful information can be concluded from this figure. First, the neural coding of different tonal working memory loads is similar. The left STG, PCG, and SMA exhibited similar neural coding for different memory loads in the encoding and maintenance phases, except for the left STG in the maintenance phase. Second, neural coding is gradually enhanced with the memory load increasing. This gradual enhancing process is easy to observe in the left STG in the encoding process and in the left PCG and SMA in both the encoding and maintenance phases. Third, the neural coding of different memory loads is different in the sensory cortex (STG). Figure 6 shows that the bilateral STG showed distinguishable neural coding for different memory loads. This finding contradicts the conclusions of some studies that neural coding in the sensory cortex is load irrelevant (Ravizza et al., 2004; Todd and Marois, 2004; Todd et al., 2005; Cowan et al., 2011; Huang et al., 2013; Majerus et al., 2016). However, some studies have also supported the view that in the sensory cortex, neural coding is load relevant (Nolden et al., 2013; Grimault et al., 2014). Finally, neural coding of tonal working memory was stored in the bilateral PCG and SMA in the maintenance phase, while neural coding in the bilateral STG disappeared after 10 s (shown in Figures 3, 4, 6, 8). This finding indicated the encoding role of the STG and the maintenance role of the PCG and SMA in tonal working memory, which suggests a hierarchical model for tonal working memory. In an mice experiment (Yu et al., 2021), it was found that optogenetic suppression of neural activity in auditory cortex during the stimulus epoch and early delay period impaired auditory working memory performance, whereas suppression later in the delay period did not. In an auditory delay-match-to-sample experiment of monkeys, (Bigelow et al., 2014) found that the firing rate of the primary auditory cortex increased only when the sound was perceived, but not during the retention phase. In an meta-analysis study (Kim, 2019) of working memory, PCG and SMA were found activated during the maintenance period. Besides, when studying neural bases of verbal and rhythmic working memory (Hoddinott et al., 2021), it was found that bilateral STG were activated only during the encoding period, while the SMA and PCG were activated during the maintenance period. All these findings support that tonal working memory has a hierarchical model, in which STG plays an encoding role, while PCG and SMA play a maintenance role.

## Conclusion

In this paper, we used an SC-MVPA method to reveal the neural coding difference between tonal working memory loads. We found that tonal working memory has significantly different neural coding compared with the control condition in the bilateral STG, PCG, and SMA. Beyond that, we found that the neural coding of adjacent tonal working memory loads were similar and hard to distinguish. In addition, we found that distinguishable neural coding differences existed in the bilateral STG and PCG for non-adjacent tonal working memory loads. We argue that as memory load increases, neural coding of tonal working memory is gradually enhanced. The difference in neural coding increased with the increase in the difference in memory load and thus became easy to distinguish. Finally, we found that the STG played an encoding role, while the PCG and SMA played a maintenance role in tonal working memory.

## Data availability statement

The datasets presented in this study can be found in online repositories. The names of the repository/repositories and accession number(s) can be found in the article/supplementary material.

## Ethics statement

The studies involving human participants were reviewed and approved by the local Ethics Committee of Southwest University. The patients/participants provided their written informed consent to participate in this study.

## Author contributions

QL: conceptualization, methodology, and writing—review and editing. DG, HT, and JT: investigation. All authors contributed to the article and approved the submitted version.

## Footnotes

1
https://www.adobe.com/cn/products/audition.html

2
https://www.freesurfer.net/

3
http://nifti.nimh.nih.gov

4
https://ww2.mathworks.cn/products/matlab.html
